# Local signs at insertion site and catheter-related bloodstream infections: an observational post hoc analysis using individual data of four RCTs

**DOI:** 10.1186/s13054-020-03425-0

**Published:** 2020-12-14

**Authors:** Niccolò Buetti, Stéphane Ruckly, Jean-Christophe Lucet, Lila Bouadma, Maité Garrouste-Orgeas, Carole Schwebel, Olivier Mimoz, Bertrand Souweine, Jean-François Timsit

**Affiliations:** 1 University of Paris, INSERM, IAME, 75006 Paris, France; 2 AP-HP, Infection Control Unit, Bichat- Claude Bernard University Hospital, 46 rue Henri Huchard, 75877 Paris Cedex, France; 3 Medical and Infectious Diseases Intensive Care Unit, AP-HP, Bichat-Claude Bernard University Hospital, 46 rue Henri Huchard, 75877 Paris Cedex, France; 4 Medical Unit, French British Hospital, Levallois-Perret, France; 5 Medical Intensive Care Unit, Grenoble University Hospital, Grenoble 1 University, La Tronche, France; 6 Services Des Urgences Adultes and SAMU 86, Centre Hospitalier Universitaire de Poitiers, 86021 Poitiers, France; 7 Université de Poitiers, Poitiers, France; 8 Inserm U1070, Poitiers, France; 9 Medical ICU, Gabriel-Montpied University Hospital, Clermont-Ferrand, France; 10 Infection Control Program and WHO Collaborating Centre on Patient Safety, University of Geneva Hospitals and Faculty of Medicine, Geneva, Switzerland; 11 Inserm U1039, Radiopharmaceutiques Biocliniques, Domaine de la Merci, 38700 La Tronche, France

**Keywords:** Insertion site, Exit-site, Intravascular catheter, Intravascular catheter infection, Catheter-related bloodstream infection, Local sign

## Abstract

**Background:**

Little is known on the association between local signs and intravascular catheter infections. This study aimed to evaluate the association between local signs at removal and catheter-related bloodstream infections (CRBSI), and which clinical conditions may predict CRBSIs if inflammation at insertion site is present.

**Methods:**

We used individual data from four multicenter randomized controlled trials in intensive care units (ICUs) that evaluated various prevention strategies for arterial and central venous catheters. We used multivariate logistic regressions in order to evaluate the association between ≥ 1 local sign, redness, pain, non-purulent discharge and purulent discharge, and CRBSI. Moreover, we assessed the probability for each local sign to observe CRBSI in subgroups of clinically relevant conditions.

**Results:**

A total of 6976 patients and 14,590 catheters (101,182 catheter-days) and 114 CRBSI from 25 ICUs with described local signs were included. More than one local sign, redness, pain, non-purulent discharge, and purulent discharge at removal were observed in 1938 (13.3%), 1633 (11.2%), 59 (0.4%), 251 (1.7%), and 102 (0.7%) episodes, respectively. After adjusting on confounders, ≥ 1 local sign, redness, non-purulent discharge, and purulent discharge were associated with CRBSI. The presence of ≥ 1 local sign increased the probability to observe CRBSI in the first 7 days of catheter maintenance (OR 6.30 vs. 2.61 [> 7 catheter-days], p_heterogeneity_ = 0.02).

**Conclusions:**

Local signs were significantly associated with CRBSI in the ICU. In the first 7 days of catheter maintenance, local signs increased the probability to observe CRBSI.

## Background

Infections due to central venous catheters and arterial catheters significantly increase hospitalization duration, hospital costs, patient morbidity and mortality in critically ill adult patients [1–3]. Moreover, intravascular catheter-related bloodstream infections are frequent events in the intensive care unit (ICU) setting [4]. Numerous risk factors associated with catheter-related bloodstream infections (CRBSIs) have been identified in several studies [5–8]. More specifically, in the last 20 years, only one old study assessed the exit-site signs as a predictor for intravascular catheter infections [9]. These data reflect practices from an arguably bygone era [10]. Indeed, the use of intravascular catheter bundles has recently changed the landscape of the risk factors [11] and, probably, of the clinical predictors for intravascular catheter infections. Moreover, recent studies suggested that the epidemiology of intravascular catheter-related infections is changing [12, 13]. To the best of our knowledge, there are no recent data on the role of exit-site signs with regards to intravascular catheter infections. In short-term intravascular catheters, the extraluminal route of infection (i.e., originating from the dermal surface) predominates [14]: it is conceivable that local signs at catheter insertion may be linked to intravascular short-term catheter infections. Our primary objective was to determine whether local symptoms and signs at insertion site were associated with CRBSI using data from four randomized controlled trials (RCTs). The secondary objective was to determine which clinical conditions increase the probability to observe CRBSI if inflammation at insertion site is present.

## Material and methods

### Data sources

This study included four longitudinal databases from four RCTs: DRESSING1 [15], DRESSING2 [16], ELVIS [17] and CLEAN [18]. Merging the data was facilitated by the fact that all these studies rely on the same definitions and similar inclusion criteria. These four studies had similar objectives: to assess the impact of specific prevention strategies on the rate of colonization and infection of intravascular catheters in ICU. Details on the studies are in the Additional file 1. The studies were approved by the national ethics committee. This post hoc analysis was not pre-planned. All RCTs complied with CONSORT guidelines and the current analysis complied with the STROBE guidelines for observational studies [19, 20].

### Study patients

The patients were recruited from 2006 to 2014 in various ICUs in France. The patients were included if they required a catheterization with a short-term central venous catheter, a short-term dialysis catheter, or peripheral arterial catheter (AC), with an expected duration of use of more than 48 h (see Additional file 1). The patients underwent follow-up until 48 h after ICU discharge.

### Study catheters

For the current analysis, short-term dialysis catheters were considered as central venous catheters (CVCs). All catheters in a given patient were managed in the same way, and all study centers complied with the French recommendations for catheter insertion and care, which are similar to CDC recommendations [21] (see Additional file 1). Catheters were removed if no longer needed, in the case of dysfunction or thrombosis or if an infection was suspected. Catheter tips were cultured using quantitative culture techniques.

### Definitions and evaluation criteria

Each study patient was evaluated daily by a team of research nurses. The patient was asked about discomfort at the insertion site, and the site was visually inspected for inflammation. These data were routinely collected at catheter removal. Local symptom (i.e.*,* “pain”) or signs as redness (i.e.*,* redness ≥ 0.5 cm), pain, non-purulent discharge and purulent discharge were noted as either absent or present. We mostly focused on a composite variable including “≥ 1 local signs or symptom.” For sake of simplicity, we used the term “local signs” instead of “local signs or symptom” throughout the text. Of note, the center investigators reported whether a catheter infection was subjectively suspected (“suspicion of infection”). The information on pathological body temperature (body temperature ≥ 38·5 °C or hypothermia with body temperature ≤ 36·5 °C) 24 h before catheter removal was collected and analyzed.

According to French [22] and American guidelines [23], the following definitions were used. A CRBSI (or “infected catheter”) was a combination of (outcome): (1) one or more positive peripheral blood cultures sampled 48 h before or after catheter removal; (2) isolation of the same organism (same species and same susceptibility pattern) from the colonized catheter or from the catheter insertion site, or a blood culture differential time to positivity of 2 h or more [24]; and (3) no apparent source of bacteremia other than the catheter. If a patient had a positive blood culture for coagulase-negative staphylococci (CoNS), a CoNS CRBSI was diagnosed solely if the pulsotype was the same among the strains recovered from the catheter and blood culture. Alternatively, at least two positive cultures with CoNS from separate blood samples were required. Catheter colonization was defined as a quantitative catheter tip culture yielding ≥ 1000 colony-forming units/mL [25]. For patients without catheter cultures, a masked adjudication committee determined whether bloodstream infections were classified as catheter related. The skin colonization was evaluated using semi-quantitative insertion-site cultures: the insertion site was sampled immediately before catheter removal. Because the size of the counting surface was different across studies, we created a semi-quantitative variable with sterile, low-grade colonization, and high-grade colonization according to the median of quantitative cultures obtained in each study.

### Statistical analysis

Characteristics of patients and catheters were described as count (percent) or median (interquartile range) for qualitative and quantitative variables, respectively.

The statistical plan had two steps: (1) The primary objective was to evaluate the association between local sign and CRBSI after adjusting for the other CRBSI confounders; (2) the secondary objective was to evaluate the role of the different local signs for observing CRBSI in subgroup of clinically relevant populations. Moreover, we added an explanatory analysis including skin colonization at removal.

We used univariate logistic regressions in order to identify variable associated with CRBSI, and we calculated odds ratios (ORs) for ≥ 1 local sign, redness, pain, non-purulent discharge, and purulent discharge. We then performed multivariate logistic regression forcing the “local sign” (i.e., ≥ 1 local sign, redness, pain, non-purulent discharge, and purulent discharge) variables and adjusting for the other variables associated CRBSI (i.e., outcome) in order to determine the association between local signs and CRBSI. Of note, the final choice of adjustment variables was based on the following clinical well-known risk factors for CRBSI [26]: sex, SOFA score, duration of catheter maintenance, experience of the operator, catheter type, insertion site, skin antisepsis, and antibiotic at insertion [5–7, 18, 27]. Logistic models were stratified for the different centers included in the analysis. Moreover, to evaluate the possible clustering effect of multiple catheters per patient, we performed a sensitivity analysis for the first catheter inserted in an individual patient.

The risk to observe CRBSI for the different local signs in subgroup of clinically relevant populations (i.e., suspicion of catheter infection vs. any suspicion of infection, pathological temperature within the 24 h before removal vs. physiological temperature, duration of catheter maintenance ≤ 7 days vs. > 7 days, catheter type CVC vs. AC, presence of immunosuppression or not, or SOFA score ≤ 11 points vs. > 11 points) was calculated with univariate logistic regression. The heterogeneity between each subgroup of clinically relevant populations was assessed using the Cochran's Q test.

For the explanatory analyses including skin colonization, we compared groups using Chi-square or Fisher tests, as appropriate.

All statistical analyses were performed with SAS (version 9.4) and R (Version 3.5.3) [28, 29].

## Results

### Description of patients and catheters

A total of 6976 patients and 14,590 catheters (101,182 catheter-days) from 25 ICUs with described local signs were included in this study (see Additional file 1: Figure E1): 2033 (29.1%) from the CLEAN study, 1460 (20.9%) from ELVIS, 1614 (23.1%) from DRESSING1 and 1869 (26.8%) from DRESSING2. The characteristics of the patients and catheters are illustrated in Tables 1, 2, and 3.

**Table 1:** Patients’ (*n* = 6976) characteristics

Variable	*n* (%)
Sex	
Male	4471 (64.1)
Age/median [IQR]	64 [53; 74]
Main reason for ICU admission	
Septic shock	1508 (21.6)
Scheduled surgery	237 (3.4)
Trauma	422 (6)
Multi-organ failure	215 (3.1)
Cardiogenic shock	568 (8.1)
Hemorrhagic shock	294 (4.2)
Other shock	202 (2.9)
De novo respiratory failure	1585 (22.7)
COPD exacerbation	127 (1.8)
Renal failure	533 (7.6)
Coma	662 (9.5)
Continuous surveillance	623 (8.9)
No comorbidity	4098 (58.7)
Cancer	377 (5.4)
Chronic renal failure	348 (5)
Chronic heart failure	513 (7.4)
AIDS	158 (2.3)
Immunosuppression	394 (5.6)
Solid organ transplantation	269 (3.9)
Hematologic neoplasia or metastatic cancer	353 (5.1)
Diabetes mellitus	557 (8)
Chronic respiratory failure	359 (5.1)
Mechanical ventilation at admission	5167 (74.1)
Vasopressor at admission	2548 (36.5)
SOFA score, median (IQR)	10.5 [7; 14]
Length of stay in hospital, median (IQR)	24 [12; 46]
ICU mortality	2343 (33.6)

IQR, interquantile range; ICU, intensive care unit; AIDS, acquired immunodeficiency syndrome; SOFA, Sequential Organ Failure Assessment

**Table 2:** Catheters’ (*n* = 14,590) characteristics

Variable	*n* (%)
Catheter days, mean (SD)/median [IQR]	6.9 (6.5)/5 [2; 9]
CVC	8500 (58.3)
Experience of the operator	
Junior (< 50 procedures)	8828 (60.5)
Senior (≥ 50 procedures)	5762 (39.5)
Insertion site	
Jugular	2706 (18.5)
Subclavian	2160 (14.8)
Femoral	5795 (39.7)
Radial	3929 (26.9)
Mechanical ventilation at insertion	11,104 (76.1)
Vasopressor at insertion	7804 (53.5)
Antibiotic at insertion	9149 (62.7)

SD, standard deviation; IQR, interquantile range; CVC, central venous catheter

**Table 3:** Symptoms, local signs and outcomes at catheter removal (*n* = 14,590)

Variable	
Catheter removal for suspected infection	2034 (13.9)
Temperature ≤ 36.5 or ≥ 38.5 at removal	7979 (54.7)
Local signs or symptom	
≥ 1 local sign	1938 (13.3)
Redness	1633 (11.2)
Pain	59 (0.4)
Non-purulent discharge	251 (1.7)
Purulent discharge	102 (0.7)
Outcomes	
Catheter colonization	1186 (8.1)
CRBSI	114 (0.8)
Reason for removal	
Death	3183 (21.8)
Catheter no longer needed	4577 (31.4)
Suspicion of infection	1981 (13.6)
Exit ICU	3098 (21.2)
Symptoms or signs at removal	
Catheter removal for suspected infection	2034 (13.9)
Temperature ≤ 36.5 or ≥ 38.5 at removal	7979 (54.7)

CRBSI, catheter-related bloodstream infection

There were 8500 (58.3%) CVCs and 6090 (41.7%) ACs.

Overall, 13.9% (2034) catheters were removed for suspected infection, whereas pathological body temperature was present in 54.7% (7979) of catheters at removal. At least one local sign, redness, pain, non-purulent discharge, and purulent discharge were observed in 13.3% (1938), 11.2% (1633), 0.4% (59), 1.7% (251), 0.7% (102) of cases, respectively.

The incidence-density per 1000 catheter-days was 1.1 for CRBSI (0.8% of the total number of catheters, 114 events) and 11.7 for catheter colonization (8.1%, 1186).

**Fig. 1:**
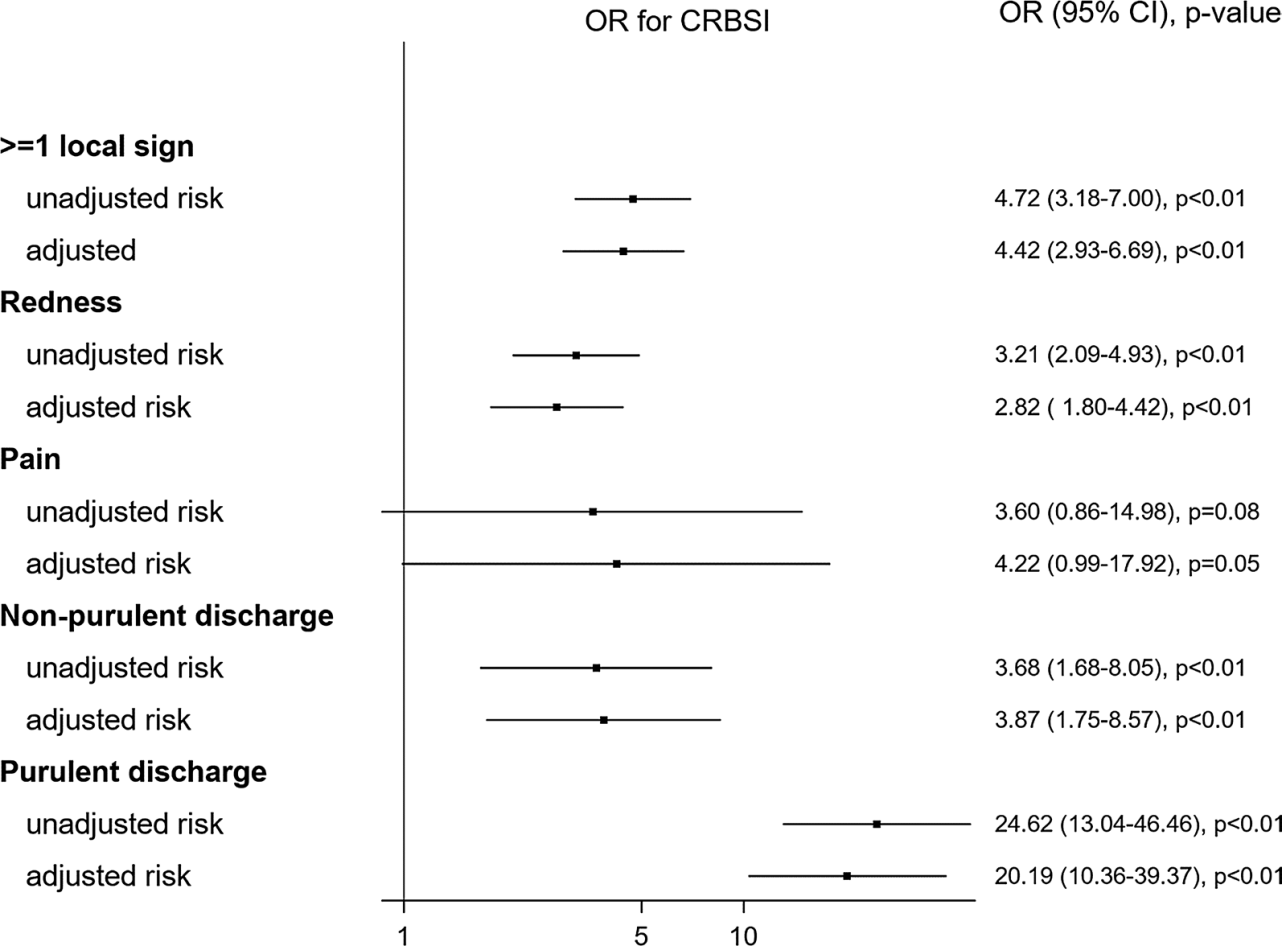
Unadjusted and adjusted local sign risk for catheter-related bloodstream infection. We adjusted for the following confounding factors for CRBSI: Sex, SOFA, catheter days, catheter type, experience of the operator, insertion site, skin antisepsis, CHG-dressing and antibiotics at insertion. OR, odds ratio; CI, confidence interval; CRBSI, catheter-related bloodstream infection

Odds ratios (ORs) for unadjusted and adjusted local signs for CRBSI are illustrated in Fig. 1.

### At least one local sign

At least one local sign was associated with CRBSI in the univariate (OR 4.72, 95% confidence interval [CI] 3.18–7.00, *p* < 0.01, Fig. 1 and Additional file 1: Table E1) and after adjusting on confounders (OR 4.42, 95% CI 2.93–6.69, *p* < 0.01, Fig. 1 and Additional file 1: Table E2). For ≥ 1 local sign, similar results were observed only when the first catheter in an individual patient was considered (OR 4.63, 95% CI 2.35–9.12, *p* < 0.01). This association remained statistically significant from 2007 to 2014 (data not shown).

**Fig. 2:**
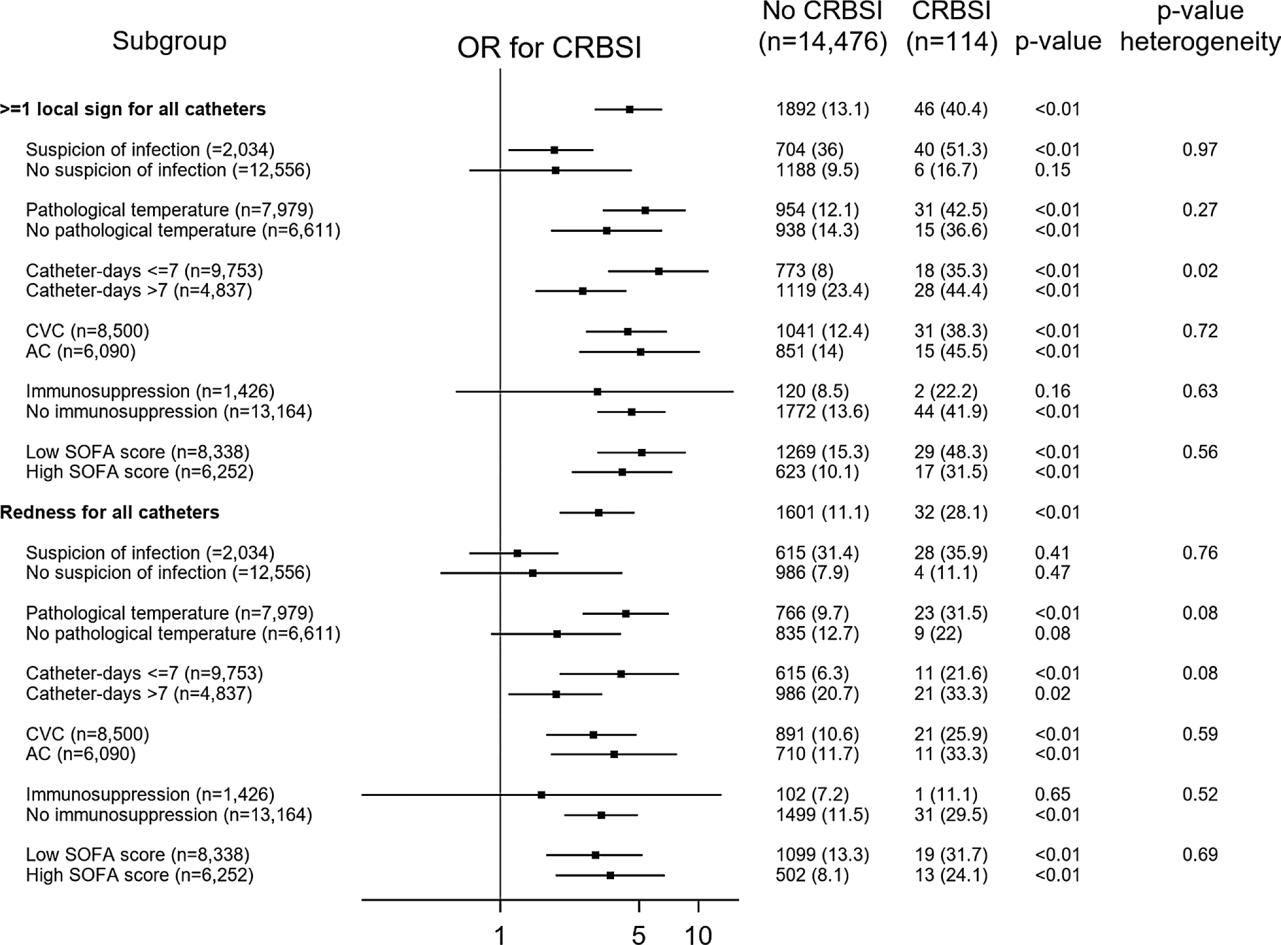
Probability to observe catheter-related bloodstream infection for the variable ≥ 1 local sign or redness in different subgroups. The group immunosuppression included AIDS patients, solid organ transplantation and other immunosuppression (see Table 1). CRBSI, catheter-related bloodstream infection (or infected catheter); CVC, central venous catheter; AC, arterial catheter; SOFA, sequential organ failure assessment. Low SOFA: ≤ 11 points. High SOFA: > 11 points

At least one sign was present in 40.4% of the infected catheters (vs. 13.1% of the non-infected catheters, *p* < 0.01, Fig. 2), and its probability to observe CRBSI was higher in the first 7 days of catheter maintenance (OR 6.30 vs. OR 2.61 for > 7 days, *p*_for heterogeneity_ = 0.02).

However, suspicion of infection (*p*_for heterogeneity_ = 0.97), pathological temperature at removal (*p*_for heterogeneity_ = 0.27), the catheter type (*p*_for heterogeneity_ = 0.72), and SOFA score (*p*_for heterogeneity_ = 0.56) did not increase the probability to observe CRBSI. We observed a non-significantly increased OR for ≥ 1 local sign (3.1, 95% CI 0.64–15.03, *p* = 0.16) in the immunosuppressed population (*n* = 1426), whereas in non-immunosuppressed patients (*n* = 13,164) a significantly increased CRBSI risk for ≥ 1 local sign was observed (OR 4.59, 95% CI 3.11–6.79, *p* < 0.01). We did not identify a significant heterogeneity between these two groups (*p*_for heterogeneity_ = 0.63).

### Redness

In the univariate logistic regression, redness was associated with CRBSI (OR 3.21, 95% CI 2.09–4.93, *p* < 0.01, Fig. 1 and Additional file 1: Table E1). After adjusting for CRBSI confounding factors, the OR for redness was 2.82 (95% CI 1.80–4.42, *p* < 0.01, Fig. 1 and Additional file 1: Table E3). Similar results for CRBSI were observed only when the first catheter in an individual patient was considered (OR 3.48, 95% CI 1.69–7.15, *p* < 0.01).

Among CRBSI, 28.1% showed redness at the insertion site (vs. non CRBSI 11.1%, *p* < 0.01, Fig. 2). Redness at insertion site was less prevalent for a catheter maintenance ≤ 7 days, but increased the probability to observe CRBSI (OR 4.06 vs. OR 1.92 [> 7 days], *p*_for heterogeneity_ = 0.08, Fig. 2). Similarly, temperature at removal showed marginal significance (*p*_for heterogeneity_ = 0.08). However, the catheter type (*p*_for heterogeneity_ = 0.59), immunosuppression (*p*_for heterogeneity_ = 0.52) and SOFA score (*p*_for heterogeneity_ = 0.66) did not increase the probability to observe CRBSI.

### Pain

In the univariate analysis, pain was not significantly associated with CRBSI (OR 3.60, 95% CI 0.86–14.98, *p* = 0.08, Fig. 1 and Additional file 1: Table E1). After adjusting for confounding factors, pain was marginally associated with CRBSI (OR 4.22, 95% CI 0.99–17.92, *p* = 0.05, Fig. 1 and Additional file 1: Table E4). No heterogeneity was observed between specific subgroups in predicting CRBSI (data not shown).

### Non-purulent discharge

In the univariate analysis, non-purulent discharge was associated with CRBSI (OR 3.68, 95% CI 1.68–8.05, *p* < 0.01, Fig. 1 and Additional file 1: Table E1). After adjusting for confounding factors, the OR for non-purulent discharge was 3.87 (95% CI 1.75–8.57, *p* < 0.01, Fig. 1, and Additional file 1: Table E5). Non-purulent discharge at insertion for a catheter maintenance ≤ 7 days increased the probability to observe CRBSI (OR 7.37 vs. OR 1.49 for > 7 days, *p*_for heterogeneity_ = 0.07, Additional file 1: Figure E2).

### Purulent discharge

In the univariate analysis, purulent discharge was associated with CRBSI (OR 24.62, 95% CI 13.04–46.46, *p* < 0.01, Fig. 1, and Additional file 1: Table E1). After adjusting for confounding factors, purulent discharge was associated with CRBSI (OR 20.19, 95% CI 10.36–39.37, *p* < 0.01, Fig. 1, and Additional file 1: Table E6). Purulence was more frequently observed in infected catheters (11.4% vs. 0.6% of non-infected catheters, *p* < 0.01, Additional file 1: Figure E2).

### Sensitivity, specificity, positive predictive value (PPV), negative predictive value (NPV), positive and negative likelihood ratio

The sensitivity, specificity, and PPV, NPV of each local sign for predicting CRBSI are illustrated in Table 4.

**Table 4:** Sensitivity, Specificity, positive predictive value, negative predictive value, positive and negative likelihood ratio for CRBSI (*n* = 114)

	Prevalence of local sign	Sensitivity	Specificity	PPV	NPV	Positive LR	Negative LR
≥ 1 local sign (*n* = 1938)	13.3	40.4	86.9	2.4	99.5	3.09	0.69
Redness (*n* = 1633)	11.2	28.1	88.9	2.0	99.4	2.54	0.81
Pain (*n* = 59)	0.4	1.7	99.6	3.3	99.2	4.46	0.99
Non-purulent discharge (*n* = 251)	1.7	6.1	98.3	2.8	99.3	3.64	0.95
Purulent discharge (*n* = 102)	0.7	11.4	99.4	12.7	99.3	18.55	0.99

PPV, positive predictive value; NPV, negative predictive value; LR likelihood ratio

The sensitivity for ≥ 1 local sign was 40.4%, whereas the highest specificities were observed for pain (99.6%) and purulent discharge (98.4%). PPV was low for redness (2%), pain (3%), non-purulent discharge (3%) and ≥ 1 local sign (2%), whereas PPV increased for purulent-discharge (12.7%). NPVs were high for all local signs.

Purulent discharge showed the highest positive likelihood ratio (18.55, 95% CI 10.68–32.21), whereas ≥ 1 local sign reduced the evidence for CRBSI (negative likelihood ratio 0.69, 95% CI 0.59–0.80). Among catheter removed for suspected infection (*n* = 2034), the NPV for ≥ 1 local sign was 97% (95% CI 95.97–97.91, data not shown). Interestingly, within the first 7 days of catheter maintenance, the positive likelihood ratio for redness and ≥ 1 local sign increased to 8.84 (95% CI 7.26–10.77) and 4.43 (95% CI 3.04–6.46, data not shown), respectively.

### Microorganism identified among CRBSI

CRBSIs were more frequently caused by *Enterobacteriaceae* (*n* = 25), polymicrobial (*n* = 24), *S. aureus* (*n* = 23), and non-fermenting Gram-negative bacilli (*n* = 16, Additional file 1: Table E7). Coagulase negative Staphylococci (CoNS) were observed in 15 CRBSI episodes.

### Skin colonization at catheter removal

The skin colonization at catheter removal was significantly more often colonized in case of ≥ 1 local sign (*p* < 0.01), redness (*p* < 0.01), non-purulent (*p* = 0.01), and purulent discharge (*p* < 0.01, Additional file 1: Table E8). The skin colonization did not significantly differ according to pain at removal (*p* = 0.74).

## Discussion

Through high quality data from four multi-centric RCTs and after correction for other confounders, this *post hoc* analysis showed that local signs at the exit site were clearly associated with intravascular catheter infections in both CVCs and ACs. This finding was more pronounced for redness, non-purulent discharge, and purulent discharge.

Three old studies examined the condition of the insertion site as a clinical predictor of CRBSI in adults. The first study included 169 patients, and the authors illustrated that infection was also associated with redness at the insertion site greater than 4 mm in diameter [30]. The second was a prospective, observational study that enrolled 1353 CVCs and showed that inflammation at the catheter site was absent in approximately 70% of CRBSIs [31]. To the best of our knowledge, only one observational study exhaustively explored this research question in the last 20 years [9]. In 2002, Safdar et al. showed that the insertion site appearance was not associated with catheter colonization or CVC-related bloodstream infections [9]. However, this study (1) reported old data collected from 1998 to 2000, probably reflecting the era prior to the routine implementation of infection prevention bundles; (2) included less than one-tenth of all catheters included in our post hoc analysis; (3) yielded a large proportion of CoNS among CRBSI; and (4) analyzed only CVC, without including AC. Similarly to the DRESSING1 and CLEAN trials, Safdar et al. also included patients from two RCTs [9], one evaluating chlorhexidine-gluconate sponge dressings [32] and the other evaluating chlorhexidine skin disinfection for prevention of CRBSI [33]. In contrast to Safdar et al*.*, local signs in our analysis were significantly associated with an increased risk for CRBSI. To support our findings, we found that skin colonization at catheter removal occurred more frequently if local signs were present. Moreover, we observed that an important proportion of CRBSI were due to *S. aureus* and Gram-negative bacilli, organisms that elicit more inflammation compared to CoNS which are less virulent [34] and in our study represented only 13% of CRBSI. In contrast, in the Safdar’s study, 87% of microorganisms identified were CoNS. In this context, and consistently with our findings, a change in the epidemiology of intravascular catheter infection toward lower prevalence of CoNS and increasing proportion of Gram-negative microorganisms has been documented in several recent studies [12, 13, 35]. In light of these considerations, the description of this cohort may probably better represent this issue.

Our findings have some important clinical Implications which, to date, have never been assessed. First, compared to the most recent literature available, local signs are associated with CRBSI, and their presence should elicit investigations for diagnosing potential intravascular catheter infections. Second, local signs may help clinicians in a specific clinical condition: if at least one local sign is present within the first 7 days of catheter maintenance, it further increases the probability to observe a catheter infection. Therefore, clinicians should deserve particular attention to the catheter insertion site in the first week after the insertion and a promptly catheter removal should be considered. Moreover, and not surprisingly, purulence at insertion site is a strong reason to remove a catheter. Interestingly, immunosuppression, pathological temperature at removal, catheter type, and severity of illness with the presence of local signs did not significantly help clinicians in predicting CRBSI. Moreover, the solely intuition of the physician who subjectively suspected an intravascular catheter infection in the presence of local signs did not increase the probability to observe a CRBSI. On the other hand, in catheters removed for suspected infection without any local signs, the probability to get infected remained low. An old RCT including a relatively low number of hemodynamically stable critically ill patients without proven bacteremia and local sign at insertion site illustrated that a “watchful waiting strategy” (*versus* immediate catheter removal) permitted a substantial decrease in the number of unnecessarily removed CVCs without increased morbidity [36]. In the case of suspicion of infection without any local signs, a catheter retention may be considered and would reduce unessentially removals. A post hoc analysis for this subpopulation in our cohort showed that CRBSI were not associated with increased mortality (OR 1.37, 95% CI 0.68–2.77, *p* = 0.38), thus suggesting, at least when catheters were routinely removed, a low mortality risk for this population.

Our study has important limitations. First, it was an observational study, and unmeasured factors may persist and cause residual confounding. However, we presented high quality exhaustive data that were prospectively collected by trained investigators and study monitors during all RCTs. Second, all RCTs were conducted in University-affiliated ICUs in France from 2006 to 2014 and included only selected short-term intravascular catheters, thus limiting the generalizability of our results. Third, we did not have any data reported for the variable “pain” in intubated or comatose patients at removal, thus probably leading to an underestimation of the proportion of this specific symptom. Fourth, redness was defined as ≥ 5 mm diameter: a smaller threshold compared to the IDSA guidelines which declared 2 cm as relevant for CRBSI [23]. We selected our cutoff in reason of the results of an old study which illustrated that infection was associated with redness > 4 mm in diameter [30]. Fifth, we described a large database designed to investigate the impact of certain prevention measures, and interactions may have occurred among the various study groups. Finally, PPVs of local signs were low, thus limiting the utilization of local signs to create algorithms for better decision-making in all patients with short-term intravascular catheters.

## Conclusions

Using the largest dataset ever collected from four multi-centric RCTs conducted with consistent catheter care, we showed that local signs were clearly associated with infections in short-term catheters in the ICU. In the first 7 days of catheter maintenance, local signs increased the probability to observe CRBSI.

## Supplementary Information

**Additional file 1:** Supplementary Material of “Local signs at insertion site and catheter-related bloodstream infections. A post hoc analysis using individual data of four RCTs”. Description: supplementary methods (data sources, patients and study catheters), supplementary Figures (Figure E1 flow-chart; Figure E2 Risk for developing CRBSI for non-purulent discharge and purulent discharge by different subgroups), supplementary Tables (Table E1: Univariate logistic model for catheter-related bloodstream infection, stratification by ICU; Table E2: Multivariate logistic models for CRBSI forcing the variable ≥ 1 local sign, stratification by ICU; Table E3: Multivariate logistic models for CRBSI forcing the variable redness, stratication by ICU; Table E4: Multivariate logistic models for CRBSI forcing the variable pain, stratification by ICU; Table E5: Multivariate logistic models for CRBSI forcing the variable non-purulent discharge, stratification by ICU; Table E6: Multivariate logistic model for CRBSI stratified by ICU forcing the variable purulent discharge; Table E7: Microorganism identified in CRBSI, E8 Skin colonization at catheter removal), supplementary references.

## Data Availability

The datasets used and/or analyzed during the current study are available from the corresponding author on reasonable request.

## Abbreviations

AC: Peripheral arterial catheter
CoNS: Coagulase-negative staphylococci
CRBSI: Catheter-related bloodstream infections
CVC: Central venous catheter
ICU: Intensive care unit
NPV: Negative predictive value (NPV)
OR: Odds ratio
PPV: Positive predictive value
RCT: Randomized controlled-trial
SOFA score: Sequential Organ Failure Assessment Score
