# Supplementary material for: Local signs at insertion site and catheter-related bloodstream infections: an observational post hoc analysis using individual data of four RCTs
Source: Crit Care. 2020 Dec 14;24:694. doi: 10.1186/s13054-020-03425-0 (PMC7737269; doi:10.1186/s13054-020-03425-0)
Supplement: Supplementary file 1 — Additional file 1: Supplementary Material of “Local signs at insertion site and catheter-related bloodstream infections. A post hoc analysis using individual data of four RCTs”. Description: supplementary methods (data sources, patients and study catheters), supplementary Figures (Figure E1 flow-chart; Figure E2 Risk for developing CRBSI for non-purulent discharge and purulent discharge by different subgroups), supplementary Tables (Table E1: Univariate logistic model for catheter-related bloodstream infection, stratification by ICU; Table E2: Multivariate logistic models for CRBSI forcing the variable ≥ 1 local sign, stratification by ICU; Table E3: Multivariate logistic models for CRBSI forcing the variable redness, stratication by ICU; Table E4: Multivariate logistic models for CRBSI forcing the variable pain, stratification by ICU; Table E5: Multivariate logistic models for CRBSI forcing the variable non-purulent discharge, stratification by ICU; Table E6: Multivariate logistic model for CRBSI stratified by ICU forcing the variable purulent discharge; Table E7: Microorganism identified in CRBSI, E8 Skin colonization at catheter removal), supplementary references. [file 13054_2020_3425_MOESM1_ESM.docx]

**Supplementary Material**

# Methods

Data sources

The DRESSING1 study investigated the impact of chlorhexidine-impregnated sponges and of the frequency of dressing changes for the prevention of catheter infection or colonization (1), whereas the DRESSING2 study assessed the effect of chlorhexidine-gluconate (CHG) dressing and highly adhesive dressing for preventing catheter-related infections and catheter colonization (2). The two other studies investigated the risk of infection with or without ethanol lock among DCs (ELVIS study, (3)), and the impact of skin antisepsis with CHG (*vs*. povidone iodine-alcohol [PVI]) on infection as also the role of skin scrubbing with antiseptic detergent before skin antisepsis on colonization (CLEAN study, (4)). CHG-sponges, CHG-dressing and CHG-skin antisepsis decreased the catheter infection rate; however, ethanol-lock, the frequency of dressing changes, highly adhesive dressing and scrubbing with antiseptic detergent did not impact infection and the colonization rates, respectively. Studies were not masked to ICU staff or the investigators, but were blinded to the microbiologists processing the skin and catheter cultures and to the adjudication committee. Data quality controls were ensured by study data monitoring during the data collection and before database lock. Informed consent was obtained from all individual participants included in the studies and whose decision-making capacity was intact. All RCTs complied with CONSORT guidelines and the current analysis complied with the STROBE guidelines for observational studies (5, 6). Clinical trials registered within [www.clinicaltrials.gov](http://www.clinicaltrials.gov) (NCT00417235, NCT01189682, NCT00875069, NCT01629550).

Patients

Patients were included in the DRESSING1 and DRESSING2 studies if they were aged at least 18 years and expected to require an arterial catheter (AC), central-vein catheter (CVC), or both inserted for 48 hours or longer. Patients with known allergies to chlorhexidine or transparent dressings were excluded. Eligible patients in the ELVIS study were adults (≥18 year) who required insertion of a non-tunneled, nonantimicrobial-impregnated double lumen short-term dialysis catheter (DC) with an expected duration of use longer than 48 hours. Exclusion criteria were known ethanol intolerance and pregnancy. In the CLEAN study, adult patients (≥18 years) who required at least one of an AC, DC, or CVC for 48 h or longer were included unless they had known intolerance, hypersensitivity, or contraindication to any trial drug (*i.e*., chlorhexidine); were likely to die within 48 h after admission; needed a catheter coated with antimicrobial agents; or had previously been enrolled in this trial.

Study catheters

Recommendations for catheter insertion and care (French guidelines - similar to CDC recommendations (7)): 1) maximal sterile barrier precautions (large sterile drape; surgical hand antisepsis; and mask, cap, sterile gloves, and gown); 2) the site of insertion was left to the discretion of the physician caring for the patient; 3) Alcoholic PVI solution or CHG was used for skin antisepsis at catheter insertion and during dressing changes; 4) semipermeable transparent dressing were used at all insertion sites and were changed 24 hours after catheter insertion and then every 3 or 7 days according to standard practice in each ICU (2-4) or randomization scheme (8). Leaking, soiled, or wet dressings were immediately changed. Ultrasound guidance was used at the discretion of the attending physician.

Catheters were removed if no longer needed, in the case of dysfunction or thrombosis or if an infection was suspected. All catheter tips were cultured using quantitative culture techniques. In patients who needed to keep the CVC beyond ICU discharge, paired blood samples were drawn simultaneously from the catheter hub and a peripheral vein before ICU discharge for determination of the differential time-to-positivity.

**Supplementary Figures**

Figure E1: Flow-chart.


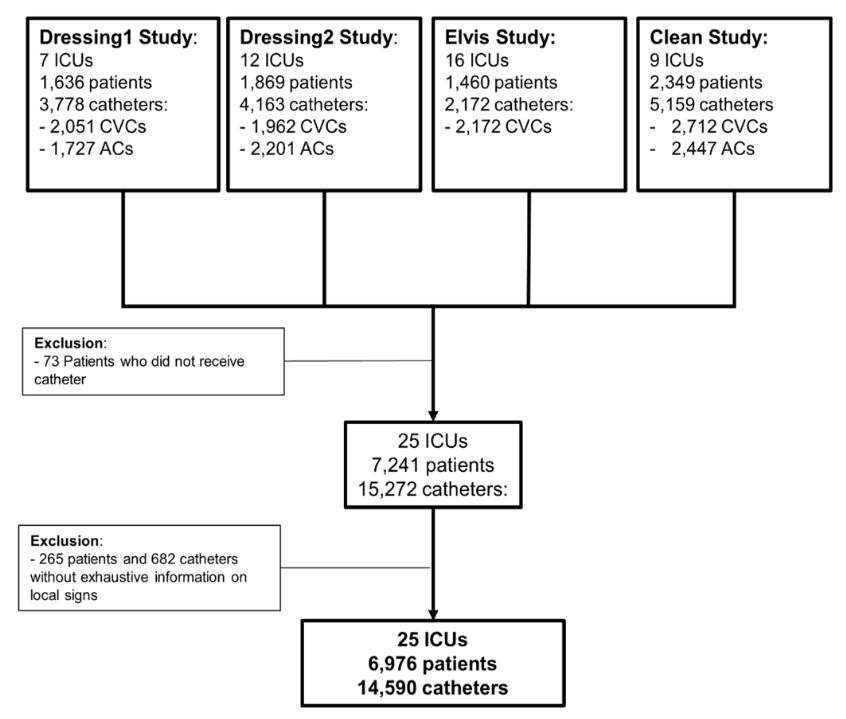


Legends. ICU: Intensive care unit. CVC: Central venous catheter. AC: Arterial catheter.

Figure E2: Risk for developing CRBSI for non-purulent discharge and purulent discharge by different subgroups.


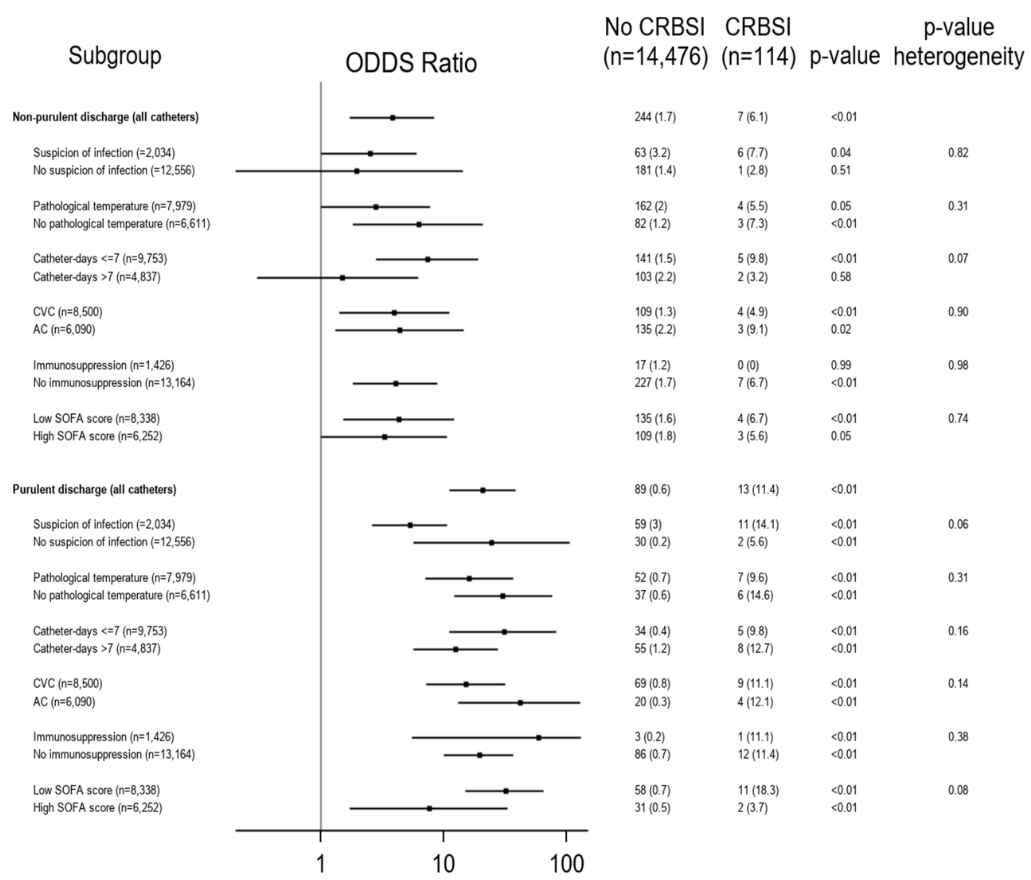


Legends. No events were observed in the CRBSI group for the group “immunosuppression”. CRBSI: Catheter-related bloodstream infection (or infected catheter). CVC: Central venous catheter. AC: arterial catheter. SOFA: Sequential Organ Failure Assessment. Low SOFA: ≤ 11 points. High SOFA: >11 points.

**Supplementary Tables**

Table E1: Univariate logistic model for catheter-related bloodstream infection, stratification by ICU

|  |  | No CRBSI | CRBSI | OR for CRBSI | 95% CI | p-value |
| --- | --- | --- | --- | --- | --- | --- |
| ≥1 local signs | | **1892 (13.1)** | **46 (40.4)** | **4.72** | **[3.18 ; 7]** | **<0.01** |
| Redness |  | **1601 (11.1)** | **32 (28.1)** | **3.21** | **[2.09 ; 4.93]** | **<0.01** |
| Pain |  | **57 (0.4)** | **2 (1.8)** | **3.60** | **[0.86 ; 14.98]** | **0.08** |
| Non-purulent discharge | | **244 (1.7)** | **7 (6.1)** | **3.68** | **[1.68 ; 8.05]** | **<0.01** |
| Purulent discharge | | **89 (0.6)** | **13 (11.4)** | **24.62** | **[13.04 ; 46.46]** | **<0.01** |
|  |  |  |  |  |  |  |
| Male sex |  | 9361 (64.7) | 61 (53.5) | 0.64 | [0.44 ; 0.92] | 0.02 |
| Age, median (IQR) | | 63 [52 ; 74] | 62.5 [53 ; 73] | 0.999 | [0.99 ; 1.01] | 0.93 |
| SOFA score, median (IQR) | | 11 [8 ; 14] | 11 [8 ; 16] | 1.04 | [1 ; 1.08] | 0.08 |
| Immunosuppression | | 1417 (9.8) | 9 (7.9) | 0.79 | [0.4 ; 1.59] | 0.51 |
| Mechanical ventilation at inclusion | | 11194 (77.3) | 81 (71.1) | 0.74 | [0.48 ; 1.13] | 0.16 |
| Vasopressor at inclusion | | 9816 (67.8) | 72 (63.2) | 0.85 | [0.57 ; 1.25] | 0.40 |
| ≤7 catheter-days | | 9702 (67) | 51 (44.7) | 0.38 | [0.26 ; 0.56] | <0.01 |
| Experience of the operator <50 procedures | | 8761 (60.5) | 67 (58.8) | 1.06 | [0.69 ; 1.64] | 0.78 |
| Catheter type - CVC (versus AC) | | 8419 (58.2) | 81 (71.1) | 1.74 | [1.14 ; 2.64] | <0.01 |
| Insertion site | Jugular | 2675 (18.5) | 31 (27.2) | 2.07 | [1.18 ; 3.64] | |
|  | Subclavian | 2145 (14.8) | 15 (13.2) | 1.21 | [0.62 ; 2.35] | |
|  | Femoral | 5749 (39.7) | 46 (40.4) | 1.36 | [0.8 ; 2.3] |  |
|  | Radial | 3907 (27) | 22 (19.3) | 1.00 |  | 0.07 |
| Skin antisepsis with CHG | | 6541 (45.2) | 45 (39.5) | 0.68 | [0.43 ; 1.08] | 0.10 |
| CHG-impregnated dressing | | 4046 (27.9) | 15 (13.2) | 0.35 | [0.2 ; 0.61] | <0.01 |
| Ethanol lock therapy | with Ethanol (versus without lock) | 1093 (7.6) | 13 (11.4) | 1.43 | [0.73 ; 2.81] | 0.30 |
| Mechanical ventilation at insertion | | 11016 (76.1) | 88 (77.2) | 1.08 | [0.69 ; 1.7] | 0.73 |
| Vasopressor at insertion | | 7744 (53.5) | 60 (52.6) | 1.01 | [0.69 ; 1.48] | 0.95 |
| Antibiotics at insertion | | 9073 (62.7) | 76 (66.7) | 1.15 | [0.77 ; 1.71] | 0.51 |

Legend. CRBSI: Catheter-related bloodstream infection. OR: Odds ratio. CI: Confidence interval. IQR: Interquartile range. ICU: Intensive care unit. SOFA: Sequential Organ Failure Assessment. CVC: Central venous catheter. AC: arterial catheter. CHG: Chlorhexidine-gluconate

Table E2: Multivariate logistic models for CRBSI forcing the variable ≥1 local sign, stratification by ICU

|  |  | OR | 95% CI | p-value |
| --- | --- | --- | --- | --- |
| ≥1 local signs | | 4.42 | [2.93 ; 6.69] | <0.01 |
| Male sex |  | 0.63 | [0.43; 0.91] | 0.01 |
| SOFA score | | 1.03 | [0.99; 1.08] | 0.15 |
| ≤7 catheter-days | | 0.51 | [0.34; 0.76] | <0.01 |
| Catheter type - CVC (versus AC) | | 1.79 | [0.88; 3.66] | 0.11 |
| Experience of the operator <50 procedures | | 0.96 | [0.63; 1.48] | 0.86 |
| Insertion site | Jugular | 1.08 | [0.44; 2.66] | 0.87 |
|  | Subclavian | 0.51 | [0.19; 1.38] | 0.18 |
|  | Femoral | 0.95 | [0.45; 2.00] | 0.89 |
| Skin antisepsis with CHG | | 0.67 | [0.43; 1.05] | 0.08 |
| CHG-impregnated dressing | | 0.35 | [0.20-0.63] | <0.01 |
| Antibiotics at insertion | | 0.99 | [0.66;1.49] | 0.97 |

Legend: CRBSI: Catheter-related bloodstream infection. OR: Odds ratio. CI: Confidence interval. IQR: Interquartile range. SOFA: Sequential Organ Failure Assessment. CVC: Central venous catheter. AC: arterial catheter. CHG: Chlorhexidine-gluconate. A sensitivity analysis for the first catheter showed similar results with OR for ≥1 local sign 4.63 (2.35-9.12, p<0.01)

Table E3: Multivariate logistic models for CRBSI forcing the variable redness, stratication by ICU

|  |  | OR | 95% CI | p-value |
| --- | --- | --- | --- | --- |
| Redness |  | 2.82 | [1.80; 4.42] | <0.01 |
| Male sex |  | 0.62 | [0.43; 0.91] | 0.01 |
| SOFA score | | 1.03 | [0.99; 1.07] | 0.20 |
| ≤7 catheter-days | | 0.45 | [0.31; 0.68] | <0.01 |
| Catheter type - CVC (versus AC) | | 1.80 | [0.88; 3.68] | 0.11 |
| Experience of the operator <50 procedures | | 0.96 | [0.63; 1.47] | 0.85 |
| Insertion site | Jugular | 1.01 | [0.41; 2.46] | 0.99 |
|  | Subclavian | 0.50 | [0.19; 1.35] | 0.17 |
|  | Femoral | 0.93 | [0.44; 1.95] | 0.84 |
| Skin antisepsis with CHG | | 0.66 | [0.42; 1.04] | 0.07 |
| CHG-impregnated dressing | | 0.37 | [0.21; 0.65] | <0.01 |
| Antibiotics at insertion | | 1.01 | [0.67; 1.51] | 0.98 |

Legend. CRBSI: Catheter-related bloodstream infection. OR: Odds ratio. CI: Confidence interval. IQR: Interquartile range. ICU: Intensive care unit. SOFA: Sequential Organ Failure Assessment. CVC: Central venous catheter. AC: arterial catheter. CHG: Chlorhexidine-gluconate. A sensitivity analysis for the first catheter showed similar results with OR for redness 3.48 (1.69-7.15, p<0.01).

Table E4: Multivariate logistic models for CRBSI forcing the variable pain, stratification by ICU.

|  |  | OR | 95% CI | p-value |
| --- | --- | --- | --- | --- |
| Pain |  | **4.22** | **[0.99; 17.92]** | **0.05** |
| Male sex |  | 0.63 | [0.43; 0.92] | 0.02 |
| SOFA score | | 1.02 | [0.98; 1.07] | 0.27 |
| ≤7 catheter-days | | 0.38 | [0.26; 0.56] | <0.01 |
| Catheter type - CVC (versus AC) | | 1.79 | [0.88; 3.64] | 0.11 |
| Experience of the operator <50 procedures | | 0.96 | [0.63; 1.48] | 0.86 |
| Insertion site | Jugular | 0.95 | [0.39; 2.32] | 0.92 |
|  | Subclavian | 0.52 | [0.19; 1.38] | 0.19 |
|  | Femoral | 0.88 | [0.42; 1.84] | 0.73 |
| Skin antisepsis with CHG | | 0.64 | [0.40; 0.999] | 0.05 |
| CHG-impregnated dressing | | 0.37 | [0.21; 0.66] | <0.01 |
| Antibiotics at insertion | | 1.03 | [0.68; 1.55] | 0.89 |

Legend. CRBSI: Catheter-related bloodstream infection. OR: Odds ratio. CI: Confidence interval. IQR: Interquartile range. ICU: Intensive care unit. SOFA: Sequential Organ Failure Assessment. CVC: Central venous catheter. AC: arterial catheter. CHG: Chlorhexidine-gluconate. Due to the small numbers of CRBSIs we could not perform a sensitivity analysis for the first catheter for the variable pain.

Table E5: Multivariate logistic models for CRBSI forcing the variable non-purulent discharge, stratification by ICU.

|  |  | OR | 95% CI | p-value |
| --- | --- | --- | --- | --- |
| Non-purulent discharge | | **3.87** | **[1.75; 8.57]** | **<0.01** |
| Male sex |  | 0.63 | [0.43; 0.91] | 0.02 |
| SOFA score | | 1.03 | [0.98; 1.07] | 0.26 |
| ≤7 catheter-days | | 0.39 | [0.27; 0.58] | <0.01 |
| Catheter type - CVC (versus AC) | | 1.82 | [0.89; 3.70] | 0.10 |
| Experience of the operator <50 procedures | | 0.96 | [0.63; 1.47] | 0.86 |
| Insertion site | Jugular | 0.97 | [0.40; 2.36] | 0.94 |
|  | Subclavian | 0.53 | [0.20; 1.41] | 0.20 |
|  | Femoral | 0.86 | [0.41; 1.80] | 0.69 |
| Skin antisepsis with CHG | | 0.64 | [0.41; 1.01] | 0.05 |
| CHG-impregnated dressing | | 0.37 | [0.21; 0.66] | <0.01 |
| Antibiotics at insertion | | 1.01 | [0.67; 1.53] | 0.95 |

Legends. CRBSI: Catheter-related bloodstream infection. OR: Odds ratio. CI: Confidence interval. IQR: Interquartile range. ICU: Intensive care unit. SOFA: Sequential Organ Failure Assessment. CVC: Central venous catheter. AC: arterial catheter. CHG: Chlorhexidine-gluconate. A sensitivity analysis for the first catheter showed similar results with OR for non-purulent discharge 3.21 (0.73-14.13, p=0.12).

Table E6: Multivariate logistic model for CRBSI stratified by ICU forcing the variable purulent discharge

|  |  | OR | 95% CI | p-value |
| --- | --- | --- | --- | --- |
| Purulent discharge | | 20.19 | [10.36; 39.37] | <0.01 |
| Male sex |  | 0.62 | [0.43; 0.91] | 0.01 |
| SOFA score | | 1.03 | [0.98; 1.07] | 0.21 |
| ≤7 catheter-days | | 0.42 | [0.28; 0.62] | <0.01 |
| Catheter type - CVC (versus AC) | | 1.72 | [0.84; 3.51] | 0.14 |
| Experience of the operator <50 procedures | | 0.90 | [0.59; 1.40] | 0.65 |
| Insertion site | Jugular | 0.94 | [0.38; 2.31] | 0.90 |
|  | Subclavian | 0.46 | [0.17; 1.24] | 0.12 |
|  | Femoral | 0.84 | [0.40; 1.77] | 0.65 |
| Skin antisepsis with CHG | | 0.67 | [0.43; 1.07] | 0.09 |
| CHG-impregnated dressing | | 0.39 | [0.22; 0.70] | <0.01 |
| Antibiotics at insertion | | 1.04 | [0.69; 1.57] | 0.85 |

Legend. CRBSI: Catheter-related bloodstream infection. OR: Odds ratio. CI: Confidence interval. IQR: Interquartile range. ICU: Intensive care unit. SOFA: Sequential Organ Failure Assessment. CVC: Central venous catheter. AC: arterial catheter. CHG: Chlorhexidine-gluconate. A sensitivity analysis for the first catheter showed similar results with OR for purulent discharge 12.21 (3.22-46.32, p<0.01).

Table E7: Microorganism identified in CRBSI.

| Microorganism | n | % |
| --- | --- | --- |
| CoNS | 15 | 13.2 |
| Staphylococcus aureus | 23 | 20.2 |
| Enterococci | 3 | 2.6 |
| Enterobacteriaceae | 25 | 21.9 |
| Non-fermenting Gram-negative bacilli | 16 | 14 |
| Other Gram-negative | 2 | 1.8 |
| Fungi | 6 | 5.3 |
| Polymicrobial CRBSI | 24 | 21.1 |

Legend. CRBSI: Catheter-related bloodstream infection. CoNS: Coagulase-negative Staphylococci.

Table E8: Skin colonization at catheter removal.

|  |  |  | p-value |
| --- | --- | --- | --- |
|  | **without local signs** | **≥1 local signs** |  |
| High-grade colonization | 2158 (27.9) | 582 (40) | <0.01 |
| Low-grade colonization | 2214 (28.6) | 411 (28.3) |  |
| Sterile | 3373 (43.6) | 461 (31.7) |  |
|  | **without redness** | **redness** |  |
| High-grade colonization | 2253 (28.3) | 487 (39.4) | <0.01 |
| Low-grade colonization | 2265 (28.4) | 360 (29.1) |  |
| Sterile | 3446 (43.3) | 388 (31.4) |  |
|  | **without pain** | **pain** |  |
| High-grade colonization | 2725 (29.8) | 15 (34.9) | 0.74 |
| Low-grade colonization | 2613 (28.5) | 12 (27.9) |  |
| Sterile | 3818 (41.7) | 16 (37.2) |  |
|  | **without non-purulent discharge** | **non-purulent discharge** | |
| High-grade colonization | 2666 (29.6) | 74 (39.6) | 0.01 |
| Low-grade colonization | 2578 (28.6) | 47 (25.1) |  |
| Sterile | 3768 (41.8) | 66 (35.3) |  |
|  | **without purulent discharge** | **purulent discharge** |  |
| High-grade colonization | 2700 (29.6) | 40 (58.8) | <0.01 |
| Low-grade colonization | 2608 (28.6) | 17 (25) |  |
| Sterile | 3823 (41.9) | 11 (16.2) |  |

**Supplementary references**

1. Timsit JF, Schwebel C, Bouadma L, Geffroy A, Garrouste-Orgeas M, Pease S, Herault MC, Haouache H, Calvino-Gunther S, Gestin B, Armand-Lefevre L, Leflon V, Chaplain C, Benali A, Francais A, Adrie C, Zahar JR, Thuong M, Arrault X, Croize J, Lucet JC. Chlorhexidine-impregnated sponges and less frequent dressing changes for prevention of catheter-related infections in critically ill adults: a randomized controlled trial. *JAMA* 2009; 301: 1231-1241.

2. Timsit JF, Mimoz O, Mourvillier B, Souweine B, Garrouste-Orgeas M, Alfandari S, Plantefeve G, Bronchard R, Troche G, Gauzit R, Antona M, Canet E, Bohe J, Lepape A, Vesin A, Arrault X, Schwebel C, Adrie C, Zahar JR, Ruckly S, Tournegros C, Lucet JC. Randomized Controlled Trial of Chlorhexidine Dressing and Highly Adhesive Dressing for Preventing Catheter-related Infections in Critically Ill Adults. *Am J Respir Crit Care Med* 2012; 186: 1272-1278.

3. Souweine B, Lautrette A, Gruson D, Canet E, Klouche K, Argaud L, Bohe J, Garrouste-Orgeas M, Mariat C, Vincent F, Cayot S, Cointault O, Lepape A, Guelon D, Darmon M, Vesin A, Caillot N, Schwebel C, Boyer A, Azoulay E, Bouadma L, Timsit JF. Ethanol lock and risk of hemodialysis catheter infection in critically ill patients. A randomized controlled trial. *Am J Respir Crit Care Med* 2015; 191: 1024-1032.

4. Mimoz O, Lucet JC, Kerforne T, Pascal J, Souweine B, Goudet V, Mercat A, Bouadma L, Lasocki S, Alfandari S, Friggeri A, Wallet F, Allou N, Ruckly S, Balayn D, Lepape A, Timsit JF, investigators Ct. Skin antisepsis with chlorhexidine-alcohol versus povidone iodine-alcohol, with and without skin scrubbing, for prevention of intravascular-catheter-related infection (CLEAN): an open-label, multicentre, randomised, controlled, two-by-two factorial trial. *Lancet* 2015; 386: 2069-2077.

5. Eaton LA. CONSORT Guidelines. In: Gellman MD, Turner JR, editors. Encyclopedia of Behavioral Medicine. New York, NY: Springer New York; 2013. p. 486-487.

6. von Elm E, Altman DG, Egger M, Pocock SJ, Gotzsche PC, Vandenbroucke JP, Initiative S. Strengthening the Reporting of Observational Studies in Epidemiology (STROBE) statement: guidelines for reporting observational studies. *BMJ* 2007; 335: 806-808.

7. O'Grady NP, Alexander M, Burns LA, Dellinger EP, Garland J, Heard SO, Lipsett PA, Masur H, Mermel LA, Pearson ML, Raad, II, Randolph AG, Rupp ME, Saint S, Healthcare Infection Control Practices Advisory C. Guidelines for the prevention of intravascular catheter-related infections. *Clinical infectious diseases : an official publication of the Infectious Diseases Society of America* 2011; 52: e162-193.

8. Timsit JF, Schwebel C, Bouadma L, Geffroy A, Garrouste-Orgeas M, Pease S, Herault MC, Haouache H, Calvino-Gunther S, Gestin B, Armand-Lefevre L, Leflon V, Chaplain C, Benali A, Francais A, Adrie C, Zahar JR, Thuong M, Arrault X, Croize J, Lucet JC, Dressing Study G. Chlorhexidine-impregnated sponges and less frequent dressing changes for prevention of catheter-related infections in critically ill adults: a randomized controlled trial. *Jama* 2009; 301: 1231-1241.
